# White matter microarchitecture and structural network integrity correlate with children intelligence quotient

**DOI:** 10.1038/s41598-020-76528-x

**Published:** 2020-11-26

**Authors:** Ilaria Suprano, Gabriel Kocevar, Claudio Stamile, Salem Hannoun, Pierre Fourneret, Olivier Revol, Fanny Nusbaum, Dominique Sappey-Marinier

**Affiliations:** 1 CREATIS, CNRS UMR 5220, INSERM U1206, INSA-Lyon, Université de Lyon, Université Claude Bernard-Lyon 1, Villeurbanne, France; 2 Nehme and Therese Tohme Multiple Sclerosis Center, Faculty of Medicine, American University of Beirut, Beirut, Lebanon; 3 Service de Psychopathologie du développement de l’Enfant et de l’Adolescent, Hospices Civils de Lyon, Lyon, France; 4 Laboratoire Parcours Santé Systémique (EA 4129), Centre PSYRENE, Université de Lyon, Université Claude Bernard-Lyon 1, Villeurbanne, France; 5 CERMEP - Imagerie du Vivant, Université de Lyon, Villeurbanne, France

**Keywords:** Cognitive neuroscience, Human behaviour

## Abstract

The neural substrate of high intelligence performances remains not well understood. Based on diffusion tensor imaging (DTI) which provides microstructural information of white matter fibers, we proposed in this work to investigate the relationship between structural brain connectivity and intelligence quotient (IQ) scores. Fifty-seven children (8–12 y.o.) underwent a MRI examination, including conventional T1-weighted and DTI sequences, and neuropsychological testing using the fourth edition of Wechsler Intelligence Scale for Children (WISC-IV), providing an estimation of the Full-Scale Intelligence Quotient (FSIQ) based on four subscales: verbal comprehension index (VCI), perceptual reasoning index (PRI), working memory index (WMI), and processing speed index (PSI). Correlations between the IQ scores and both graphs and diffusivity metrics were explored. First, we found significant correlations between the increased integrity of WM fiber-bundles and high intelligence scores. Second, the graph theory analysis showed that integration and segregation graph metrics were positively and negatively correlated with WISC-IV scores, respectively. These results were mainly driven by significant correlations between FSIQ, VCI, and PRI and graph metrics in the temporal and parietal lobes. In conclusion, these findings demonstrated that intelligence performances are related to the integrity of WM fiber-bundles as well as the density and homogeneity of WM brain networks.

## Introduction

The investigation of the neural substrate of intelligence has been a topic of increasing interest in the neuroscience field. Indeed, based on the Jung and Haier meta-analysis review of 37 neuroimaging studies, a distributed brain network, mainly based on frontoparietal connections, was identified and associated to high intelligence quotient (IQ)^1^. More recently, this model was extended to other brain regions including the posterior cingulate cortex and subcortical structures^2^. Additionally, diffusion tensor imaging (DTI) application in childhood research studies, correlated IQ scores to fractional anisotropy (FA) and mean diffusivity (MD) metrics in several WM regions^3^. Indeed, Navas-Sanchez et al. reported a significant positive correlation between IQ and FA, mainly in the corpus callosum, supporting the idea that efficient information transfer between hemispheres is crucial for higher intellectual capabilities^4^. Tamnes et al. confirmed that verbal and performance abilities were positively related to FA and that WM development was accelerated in late childhood of subjects with high VCI compared to subjects with average verbal abilities^5^. Exploring different cognitive performances, Muetzel et al. showed also that visuo-spatial abilities were related to high FA values^6^. Nevertheless, these relationships between WM integrity and IQ may depends on the gender^7^ as well as on the intelligence profile^8^. In summary, these studies paved the way of a strong relationship between WM integrity and high cognitive performance.

Furthermore, the human brain can be described as a network with “small-world” characteristics using graph theory^9^. Considering gray matter (GM) regions as brain networks nodes and WM fibers, derived from diffusion MRI tractography as links connecting the nodes, local and global metrics derived from graph theory can be measured^10^. A few studies investigated the role of structural connectivity in human intelligence. Li et al. were the first to demonstrate the association between the level of intelligence and the efficiency of the brain structural network in adults^11^. In children, two recent studies explored the relationship between graph metrics and intelligence showing some specific correlations between visual construction ability and IQ scores with structural efficiency^12,13^. Finally, brain structural connectivity was recently demonstrated to correlate particularly with fluid intelligence^14^.

In the present study, we propose to explore the neural substrate of intelligence in a cohort of children. We tested the hypothesis that children intelligence is associated with a better structural connectivity and a specific network organization at both local and global scales. To this end, the relationships between all IQ indices and topological properties of brain structural networks as well as diffusivity metrics of the main WM fiber-bundles were investigated.

## Material and methods

### Subjects

This prospective MRI study included 57 children (14 girls and 43 boys, age (mean ± SD): 9.81 ± 1.16 years). Subjects were recruited from the PSYRENE center and the children’s psychiatry department of Lyon’s Neurological Hospital as well as through advertisement in medical practices and public institutions. All children with any neurological diseases, medical psychiatric comorbidities, learning disabilities, psychotropic treatments, or contra-indications for MRI were excluded (reported in Kocevar et al.^14^). Ethical committee approval called in French “Comité de Protection des Personnes (CPP) Sud-Est IV” and written informed consent from the children and their parents were obtained.

### Intelligence scores

All children underwent the Wechsler intelligence scale for children (WISC-IV) to obtain a reliable estimation of the full-scale IQ (FSIQ). The WISC-IV is based on four subscales, namely, the verbal comprehension index (VCI), the perceptual reasoning index (PRI), the working memory index (WMI), and the processing speed index (PSI).

### MRI acquisition and processing

MRI examinations were performed on a 1.5 T Siemens Sonata system (Erlangen, Germany) with an 8-channels head-coil and 40 mT/m gradients at the MRI department of “CERMEP-Imagerie du Vivant”. As reported in Kocevar et al.^14^, the MRI protocol included a 3D T1-weighted magnetization prepared rapid gradient echo (MPRAGE) sequence (time of repetition/time of echo/time for inversion [TR/TE/TI] = 1970/3.93/1100 ms; flip angle = 15°; matrix size = 256 × 256; field of view (FOV) = 256 × 256 mm; slice thickness = 1 mm; voxel size = 1 × 1 × 1 mm; acquisition time = 8 min). DTI protocol was based on a 2D multi-slice spin-echo echo-planar imaging (EPI) sequence (TR/TE = 6900/86 ms, matrix size = 96 × 96, FOV = 240 × 240 mm, acquisition time = 7 min). Fifty-one contiguous axial slices of 2.5 mm thickness were acquired in the anterior commissure–posterior commissure (AC–PC) plane. Twenty-four diffusion gradient directions (b = 1000 s/mm^2^) were applied with a nominal isotropic resolution of 2.5 mm^3^. The b0 image was acquired four times to increase signal to noise ratio while the other directions were acquired twice.

Diffusion data preprocessing was performed using the Functional Magnetic Resonance Imaging of the Brain (FMRIB) Software Library (FSL)^15^. First, data were corrected for subjects’ motion and Eddy currents and non-brain voxels were removed using FSL-BET. From transformation matrices generated during the Eddy current correction, we obtained a mean patient displacement value of 1.26 ± 0.52 mm (mean ± SD), which is half of a voxel size (2.5 mm). For each subject, the tensor model was then fitted on diffusion data using FSL-FDT^16^ and four diffusion maps (FA, MD, AD, and RD) were extracted.

### Fiber-bundle analysis

Based on the Illinois Institute of Technology (IIT) atlas (see Supplementary Material), large WM fiber-bundles were defined including the forceps major (Fmajor), the forceps minor (Fminor), the superior and inferior longitudinal fasciculus (SLF and ILF respectively), the inferior fronto-occipital fasciculus (IFOF), the uncinate fasciculus (Unc), the cingulum (Cing), and the cortico-spinal tract (CST)^17^.

The IIT atlas FA map was co-registered to each subjects’ FA image using a non-rigid transformation performed with NiftyReg^18^ and the deformation field was applied to all fiber-bundles. Since fiber-bundles masks of IIT atlas contained the probability of each voxel to belong to a specific fiber-bundle, a threshold of 35% was used to discard all voxels having a lower probability to belong to the fiber-bundle of interest and the resulting masks were binarized. Finally, the mean value of FA, MD, AD, and RD, were extracted from each subject’s fiber-bundles^14^.

### Graphs analysis

Desikan cortical and subcortical GM parcellation (see Supplementary Material) was transformed to each subject space using the previously generated deformation field, and 84 nodes were defined^19^.

The pipeline for graph generation started from the whole brain tractography that was performed for every subject using MRtrix^20^. The main diffusion directions were estimated in each voxel using diffusion orientation distribution function (dODF) using a maximum spherical harmonics order $h=4$ to match with the acquisition protocol. Anatomically constrained probabilistic streamline tractography was then performed to generate 1,000,000 streamlines based on the four-tissue-class classification (WM, cortical GM, sub-cortical GM and cerebro-spinal fluid (CSF)) of the IIT atlas and dODF. Finally, summing the number of streamlines connecting each pair of nodes, adjacency matrices were generated for each subject. In order to remove the weakest connections generated by tractography, a proportional threshold (τ = 0.25) was applied to obtain binary connectivity matrices. This threshold, corresponding the minimum mean variability, was optimized after the estimation of the inter-subject variability of the global metrics measured for different thresholds varying between 0 and 1 in subjects with standard IQ.

Brain structural connectivity was analyzed globally as well as locally by dividing the connectivity matrices in different sub-graphs: the left and right hemispheres, and the inter-hemisphere connections graph. The connectivity matrices were also divided into lobes (frontal, occipital, parietal and temporal), cingulate, and sub-cortical GM.

Small-world properties of each subject’s graph were verified and the topological properties of the brain networks were analyzed using six global and four nodal metrics from graph theory^21^. At a global scale, the graph density ($D$) is measured as the ratio between the number of effective connections in the graph (*l*) to the number of possible connections. On one hand, the integration property of the graph was evaluated using characteristic path length (*CPL*)*,* which is the mean of the shortest paths in the graph, and *E*_*g,*_ which is the mean of the inverse of the distance matrix of the graph. On the other hand, the segregation property of the graph was analyzed using the transitivity (*T*) which is the ratio between the number of triangles and the number of triplets in the graph, the assortativity ($r$) which is the Pearson coefficient between the degrees of two nodes at the extremities of an edge, and the modularity (*Q*) which is the difference between the number of intra-modules connections and the number of inter-modules connections. At a nodal scale, the degree (*k*_*i*_) represents the number of connections of each node. The clustering coefficient (*C*_*i*_) defined as the ratio between the number of triangles and the number of triplets around each node*,* measures the network’s tendency to form dense local clusters. The betweenness centrality (*B*_*i*_), defined as the ratio of the number of the shortest paths comprising the node to the total number of shortest paths in the graph, measures the hub property of the node. The nodal efficiency (*E*_*i*_) defined as the mean of the inverse of the distance vector, measures the signal transmission efficiency.

All the metrics, except graph density, were computed based on the binarized connectivity matrices using the brain connectivity toolbox on Matlab^21^.

### Statistical analysis

Partial correlations between intelligence scores and network measures were calculated in the total sample using age and gender as covariates. A general linear model (GLM) was used to investigate the relationship between each WISC (*W*_*i*_) index and the global and nodal graph metrics as well as the diffusion metrics of the WM fiber-bundles. The general expression of this model was as follows:

$$Wi \sim metric \left(Diffusivity \,or \,Graph\right)+Age+Gender$$

Statistical significance of all predictor was tested for each fit by applying analysis-of-variance and analysis-of-deviance with a 5% significance level. All statistical analysis were computed using R^22^. In order to correct for multiple comparisons, FDR correction was applied to *p* values.

### Ethics approval

Following the ethical standards laid down in the 1964 Declaration of Helsinki, its later amendments, and in application of article L 1121-4 of the French Public Health Code, this biomedical research has obtained the approval of the local Ethic Committee named “Comité de Protection des Personnes (CPP) Sud-Est IV” and the authorization from the French medical authorities (Agence Nationale de sécurité et du Médicament (ANSM)). The written informed consent was obtained from the children and their two parents. In the consent form, both parents and the child agree that the publication of the results will be done anonymously, without any individual identifying results.

## Results

### Fiber-bundles analysis

In each WM fiber-bundle, FA and AD values were obtained and their association with intelligence scores analyzed as reported in Table 1. Positive correlations were found in several WM fiber-bundles. FA was significantly correlated with FSIQ and VCI in the Fminor bundle of the corpus callosum, and only for the FSIQ in the Fmajor bundle. AD was also significantly correlated with FSIQ and VCI, as well as PRI and WMI in both the Fmajor and Fminor bundles. In all associative fiber-bundles, except the cingulum, FA and AD metrics were correlated with intelligence scores. Significant correlations were observed between FSIQ and FA values of the left and right IFOF, and Unc fasciculi, and only with the left ILF and SLF. VCI and FA values were also correlated in the left and right IFOF. Significant correlations were observed between FA and PRI values in the left and right Unc fasciculi and left IFOF. Between PSI and FA, we found significant correlations in the left ILF. AD was significantly correlated with FSIQ, VCI, and PRI in bilateral Unc and with FSIQ and VCI in bilateral IFOF. Finally, FSIQ, VCI were correlated with AD in the right CST, while in the left CST, only VCI was correlated with AD. Significant effect for gender was found in few correlations between diffusivity metrics and WISC scores. However, this effect observed in only 5 fiber bundles, remained much lower than the diffusivity metric effect. No RD and MD correlation survived after multicomparison correction.

**Table 1:** Standardized beta and statistical significances obtained with a GLM model between the mean fractional anisotropy (FA) and axial diffusivity (AD) of the WM fiber-bundles (forceps major (Fmajor), forceps minor (Fminor), cortico-spinal tract (CST), superior and inferior longitudinal fascicle (SLF and ILF respectively), uncinate fascicle (Unc), and inferior fronto-occipital fascicle (IFOF)) and the IQ scores (full scale intelligence quotient (FSIQ), verbal comprehension index (VCI), perceptual reasoning index (PRI), working memory index (WMI), and processing speed index (PSI)).

	FSIQ		VCI		PRI		WMI		PSI	
	FA	AD	FA	AD	FA	AD	FA	AD	FA	AD
Fmajor	0.333*	0.519***	–	0.446***	–	0.337*	–	0.436**	–	–
Fminor	0.337*	0.566***	0.379*	0.526***	–	0.404*	–	0.389*	–	–
**CST**										
L	–	–	–	0.378*	–	–	–	–	–	–
R	–	0.339*	–	0.334*	–	–	–	–	–	–
**SLF**										
L	0.372*	–	–	–	–	–	–	–	–	–
R	–	0.483***	–	0.423**	–	0.332*	–	0.485**	–	–
**ILF**										
L	0.440*	–	–	0.344*	–	–	–	–	0.414*	–
R	–	0.460***	–	–	–	0.372*	–	0.428**	–	–
**Unc**										
L	0.417*	0.401**	–	0.411**	0.437*	0.370*	–	–	–	–
R	0.355*	0.625***	–	0.521***	0.359*	0.517***	–	0.509**	–	–
**IFOF**										
L	0.507**	0.355*	0.526**	0.469**	0.373*	–	–	–	–	–
R	0.354*	0.497***	0.382*	0.430**	–	0.393*	–	0.417**	–	–

Values in bold represent correlations with a significant effect for gender.

*p < 0.05; **p < 0.01; ***p < 0.001.

### Graph analysis

#### Global graph metrics

**Figure 1:**
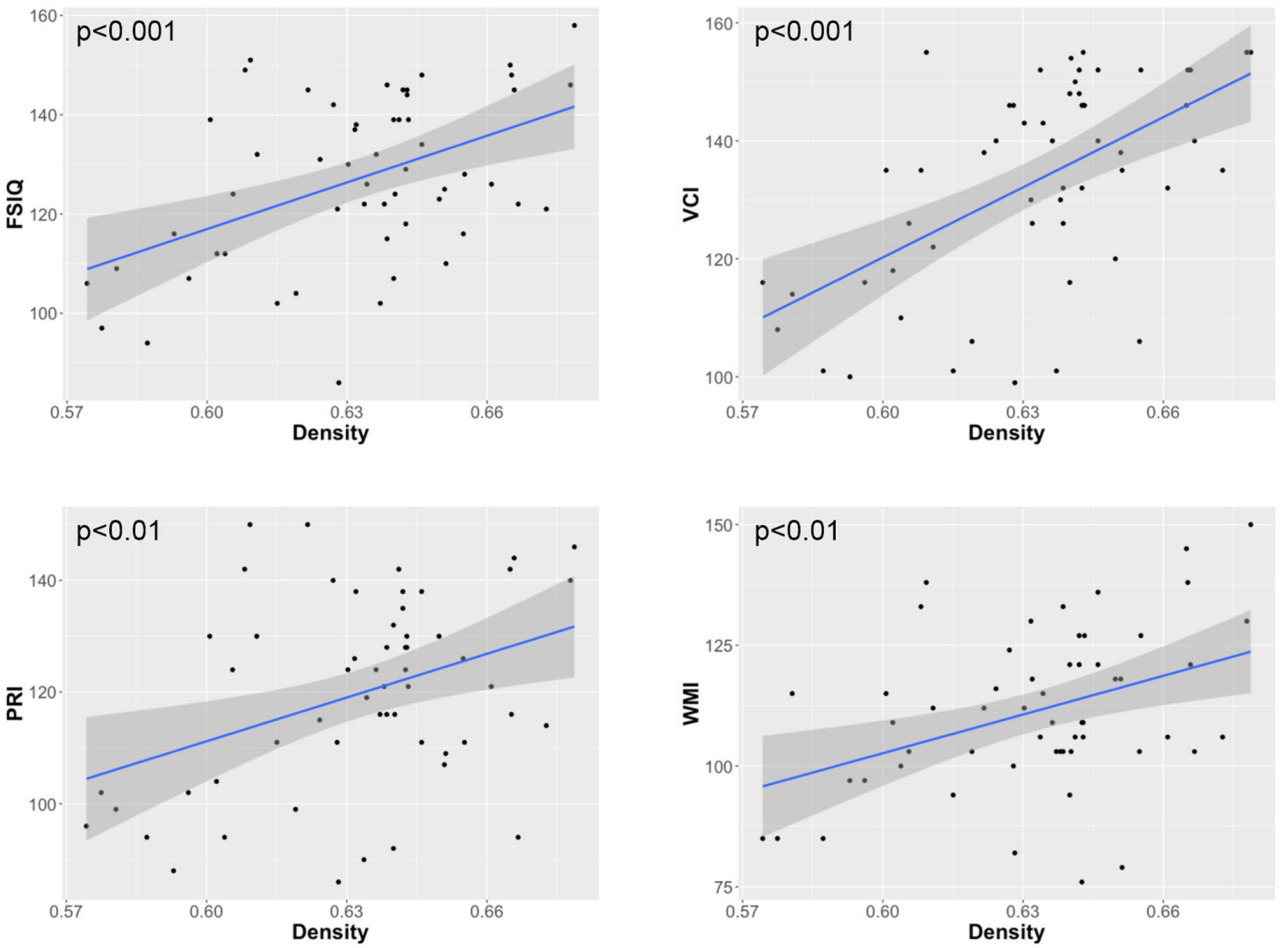
Significant positive correlations obtained between the graph density of the whole brain networks and the full-scale intelligence quotient (FSIQ), the verbal comprehension index (VCI), the perceptual reasoning index (PRI), and the working memory index (WMI).

When measured in whole brain, significant correlations were found between graph metrics and FSIQ as well as WISC-IV subscales. More in details, FSIQ, VCI, PRI, and WMI showed positive correlations with the density *D* (Fig. 1). In contrast, negative correlations were found between FSIQ, PRI, and WMI and the modularity *Q,* as well as between VCI and WMI and the transitivity *T*. When measured in both hemispheres, FSIQ, VCI, PRI, and WMI values were positively correlated with *D*. In the right hemisphere, a negative correlation was observed between PRI and *Q*. Finally, positive correlations were observed between FSIQ, VCI, and WMI and *D,* and between PSI and *T,* in the inter-hemispheric connections.

The same graph metrics were also measured in other networks such as brain lobes (frontal, parietal, occipital, and temporal) as well as sub-cortical nuclei. In the left parietal lobe FSIQ and WMI were negatively correlated with Q. In the right occipital lobe, WMI was negatively correlated with *T*, and WMI positively correlated with *CPL*. In the left temporal lobe, FSIQ and WMI were positively correlated with *r* and *T*. FSIQ, PRI, and PSI were negatively correlated with *Q*. VCI, and PRI were negatively correlated with *E*_*g*_. FSIQ, and VCI were positively correlated with CPL. Finally, in the left subcortical GM networks, PRI was negatively correlated with r, while PSI was positively correlated with T. All the correlations between global graph metrics and intelligence scores are reported in Table 2.

**Table 2:** Standardized beta and statistical significances obtained with a GLM model between global graph metrics, namely density (*D*), assortativity (*r*), transitivity (*T*), modularity (*Q*), characteristic path length (*CPL*), and efficiency (*E*_*g*_) measured in different brain networks (whole brain, inter-hemisphere, left and right hemispheres, different lobes, and subcortical regions), and the IQ scores (full scale intelligence quotient (FSIQ), verbal comprehension index (VCI), perceptual reasoning index (PRI), working memory index (WMI), and processing speed index (PSI)).

Networks		Metric	FSIQ	VCI	PRI	WMI	PSI
Whole brain	–	*D*	0.451***	0.575***	0.353**	0.421**	–
		*Q*	− 0.321*	–	− 0.281*	− 0.363*	–
		*T*	–	− 0.333 *	–	− 0.307*	–
Inter-hemisphere	–	*D*	0.374 **	0.499 ***	–	0.394**	–
		*T*	–	–	–	–	0.292*
Hemisphere	L	*D*	0.391**	0.459***	0.370**	0.362 **	–
	R	*D*	0.359**	0.436 **	0.358*	0.288*	–
		*Q*	–	–	− 0.338*	–	–
Parietal	L	*Q*	− 0.385*	–	–	− 0.437*	–
Occipital	R	*CPL*	–	–	–	0.317*	–
		*T*	–	–	–	− 0.317*	–
Temporal	L	*r*	0.282*	–	–	0.359*	–
		*Q*	− 0.385*	–	− 0.396*	–	− 0.401*
		*T*	0.298*	–	–	0.421**	–
	R	*E*_*g*_	–	− 0.421*	− 0.400*	–	–
		*CPL*	0.386*	0.440*	–	–	–
Sub-Cortical	L	*r*	–	–	− 0.301*	–	–
		*T*	–	–	–	–	0.276*

*p < 0.05; **p < 0.01; ***p < 0.001.

#### Nodal graph metrics

**Figure 2:**
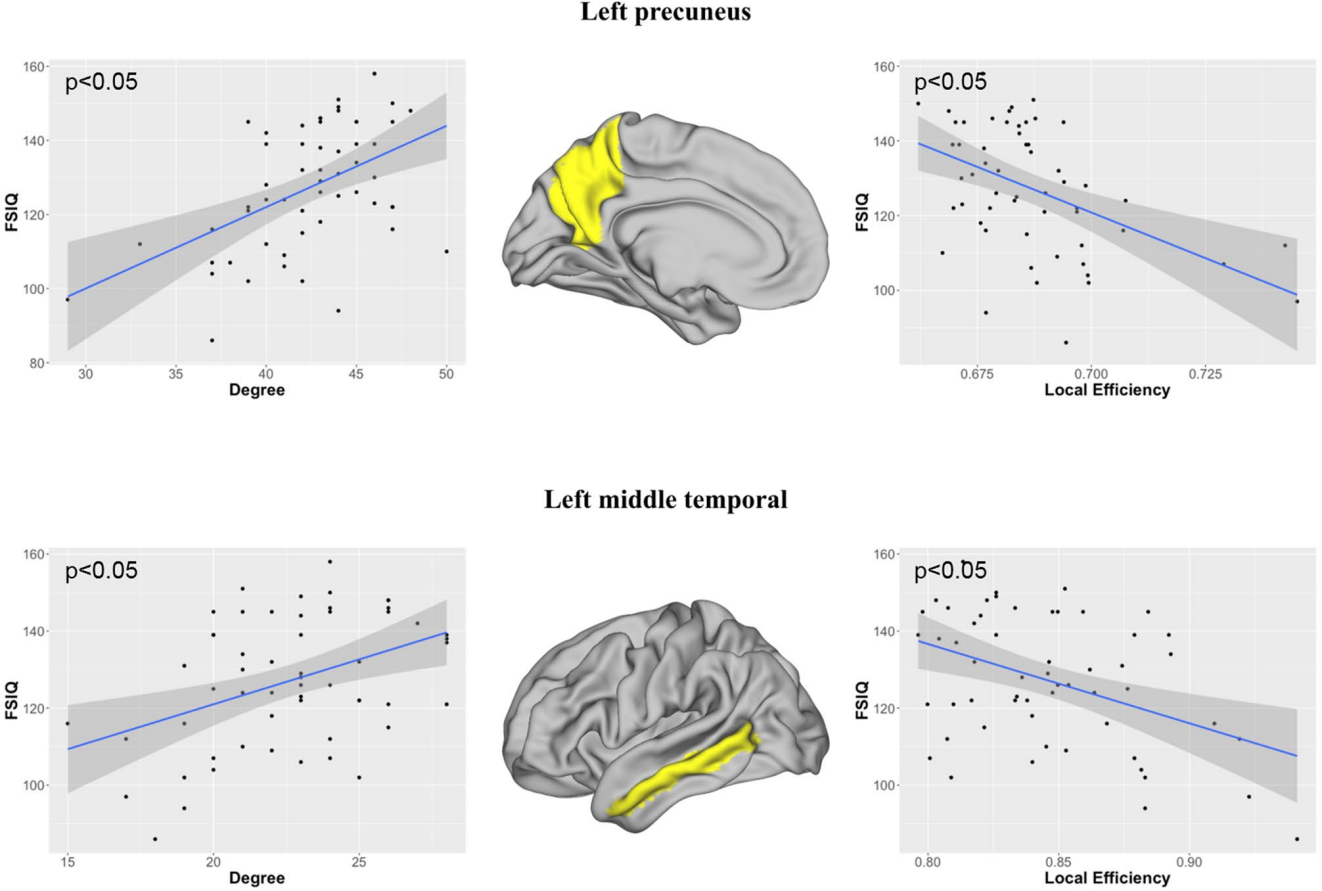
Significant correlations obtained between the full-scale intelligence quotient (FSIQ) and the local graph metrics in the left precuneus and the left middle temporal networks. Image drawn with Connectome Workbench toolbox v1.3.2 (https://humanconnectome.org/software/connectome-workbench).

**Figure 3:**
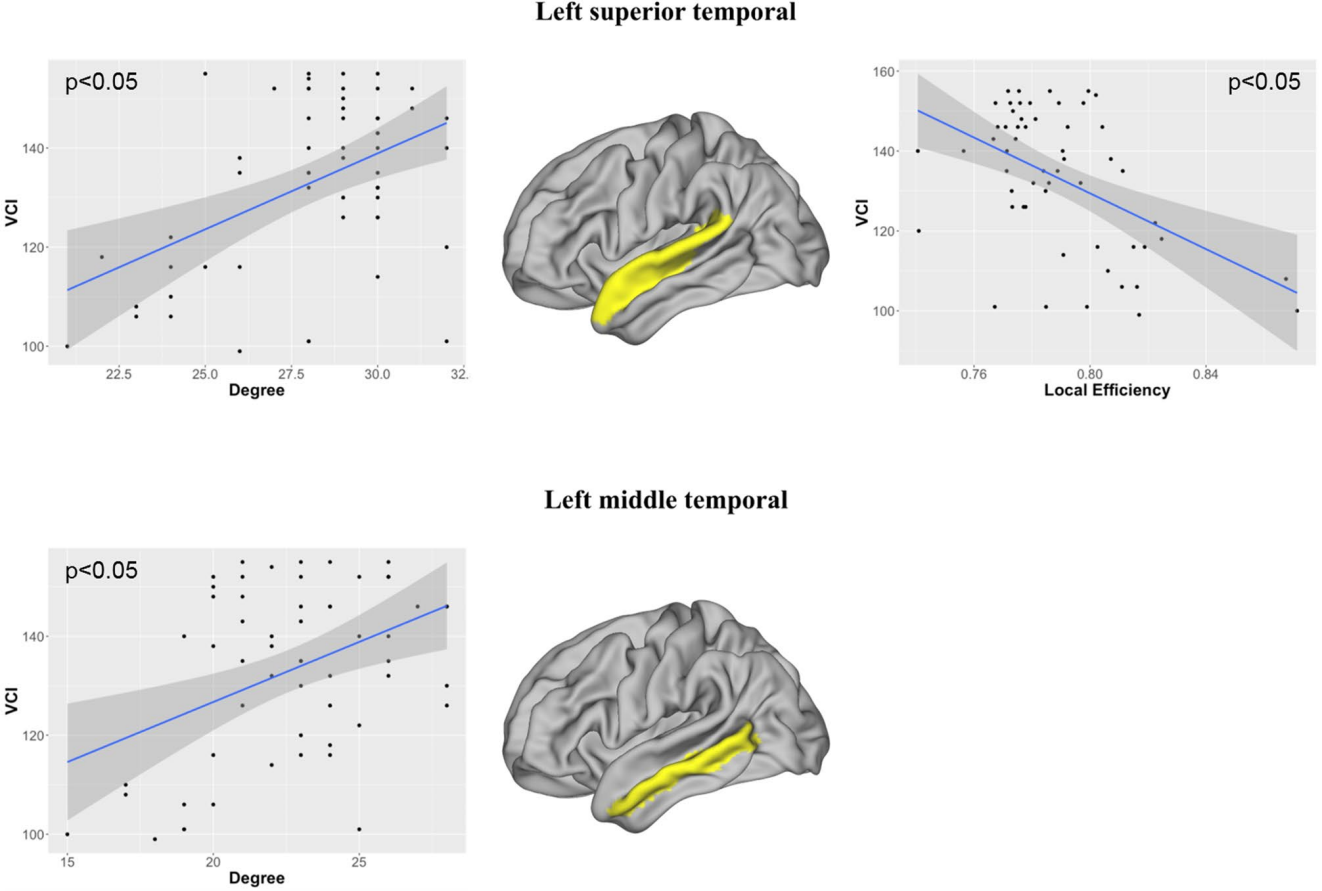
Significant correlations obtained between the verbal comprehension index (VCI) and the local graph metrics in the left superior and middle temporal networks. Image drawn with Connectome Workbench toolbox v1.3.2 (https://humanconnectome.org/software/connectome-workbench).

**Figure 4:**
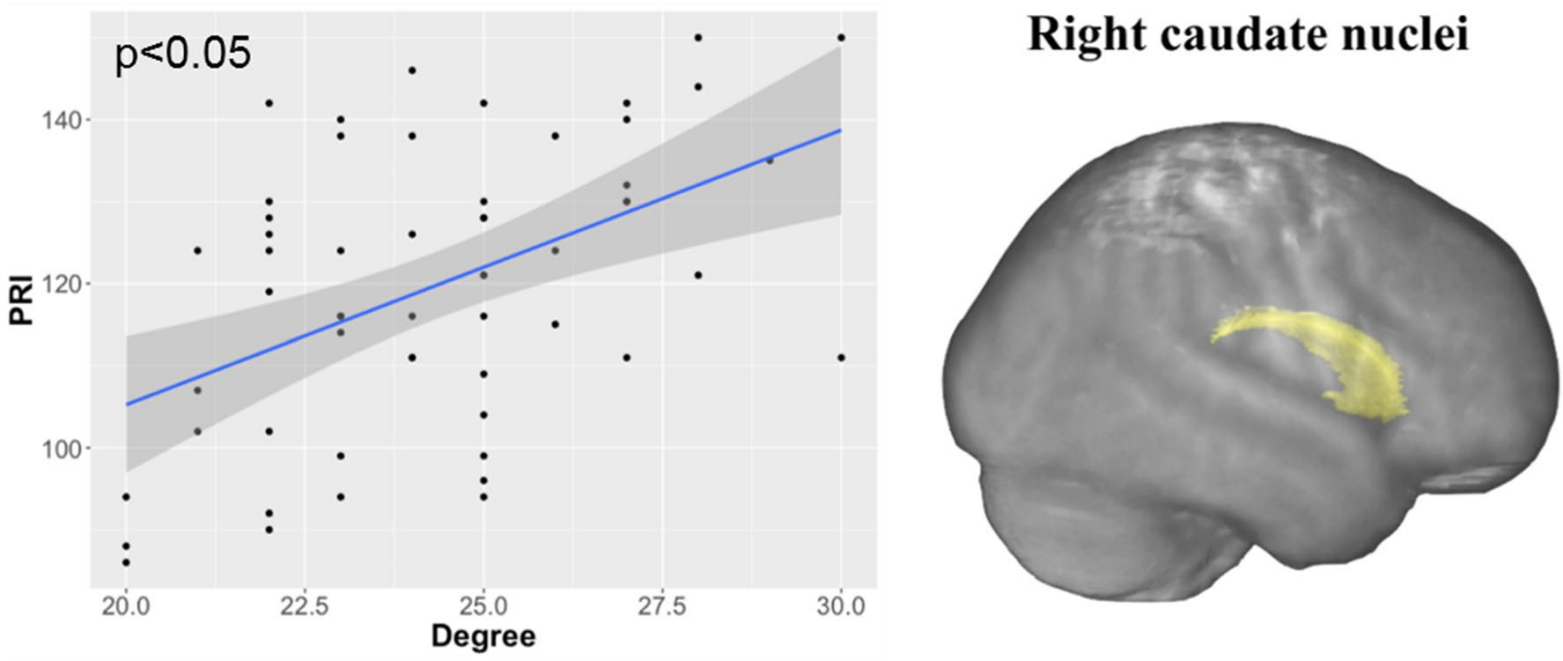
A significant positive correlation obtained between the perceptual reasoning index (PRI) and degree metric in the right caudate. Image drawn with Connectome Workbench toolbox v1.3.2 (https://humanconnectome.org/software/connectome-workbench).

Several significant correlations were observed between the nodal graph metrics and the intelligence scores, including mainly FSIQ and VCI, and to a lesser extent PRI. These correlations were observed in numerous networks, mainly located in the left hemisphere, as reported in Table 3. As shown in Fig. 2, FSIQ was positively correlated with the degree *k*_*i*_ and negatively with the local efficiency *E*_*i*_ in the left precuneus networks and in the left middle temporal networks. In the middle and superior temporal networks, VCI was positively correlated with *k*_*i*_ and only in the middle temporal networks negatively with *E*_*i*_ (Fig. 3). In the right caudate nuclei networks, PRI was positively correlated with *k*_*i*_ (Fig. 4).

**Table 3:** Standardized beta and statistical significances obtained with a GLM model between the local graph metrics, namely degree (*ki*), betweenness centrality (*Bi*), clustering coefficient (*Ci*), and efficiency (*Ei*), measured from gray matter (GM) nodes of brain lobes of each hemispheres (left (L) and right (R)), and the IQ scores (full scale intelligence quotient (FSIQ), verbal comprehension index (VCI), perceptual reasoning index (PRI), working memory index (WMI), and processing speed index (PSI)).

Networks		GM nodes	Metric	FSIQ	VCI	PRI	WMI	PSI
Frontal	L	Paracentral	*k*_*i*_	–	0.402*	–	–	–
		Frontalpole	*k*_*i*_	− 0.399*	− 0.390*	–	–	–
			*B*_*i*_	− 0.431*	–	–	–	–
Parietal	L	Precuneus	*k*_*i*_	0.464*	0.415*	0.462*	–	–
			*C*_*i*_	− 0.469*	–	− 0.484*	–	–
			*E*_*i*_	− 0.448*	–	–	–	–
	R	Supramarginal	*B*_*i*_	–	0.429*	–	–	–
Occipital	R	Lateraloccipital	*B*_*i*_	0.442*	–	–	–	–
Temporal	L	Fusiform	*k*_*i*_	–	− 0.409*	–	–	–
		Middletemporal	*k*_*i*_	0.401*	0.409*	–	–	–
			*C*_*i*_	− 0.439*	–	–	–	–
			*E*_*i*_	− 0.437*	–	–	–	–
		Superiortemporal	*k*_*i*_	–	0.451*	–	–	–
			*B*_*i*_	–	0.445*	–	–	–
			*C*_*i*_	–	− 0.482*	–	–	–
			*E*_*i*_	–	− 0.477*	–	–	–
		Transversetemporal	*k*_*i*_	− 0.473*	− 0.391*	− 0. 464*	–	–
	R	Bankssts	*k*_*i*_	–	− 0.419*	–	–	–
			*B*_*i*_	–	− 0.433*	–	–	–
		Transversetemporal	*k*_*i*_	− 0.479*	− 0.390*	− 0.430*	–	–
Sub-cortical	R	Caudate	*k*_*i*_	0.416*	0.370*	0.508**	–	–
Cerebellum	R	Cerebellum	*k*_*i*_	–	–	0.430*	–	–

* p <0.05; ** *p* <0.01; *** *p* < 0.001.

## Discussion

The present study used both graph theory and fiber-bundle analysis methods, to demonstrate the relationship of WM microarchitecture and structural connectivity metrics with children intelligence.

The exploration of structural connectivity using graph metrics provided evidence of a strong relationship between brain networks connectivity and intelligence. More specifically, we showed that higher intelligence is related to a dense and homogeneous brain networks. Indeed, most of the IQ indices (VCI, PRI and WMI) correlated positively with graph density and negatively with modularity and transitivity, two metrics describing segregation properties. These results are in accordance with the idea that intelligence depends on the brain networks capacity to enhance their structural connectivity^23^.

When analyzing the relationship between intelligence scores and graph metrics in cerebral lobes, most of the significant correlations were found in the temporal and parietal lobes, two important regions of the networks proposed by Jung and Haier in their P-FIT theory^1^. On one hand, the strong correlations observed between both VCI and WMI scores and graph metrics in the temporal lobe confirmed that high integration and density levels of the temporal connectivity are related to high verbal and memory abilities, as previously reported^24,25^. On the other hand, the high levels of structural connectivity observed in the parietal lobe in relation with high FSIQ confirmed the important role of parietal functions in children intelligence, such as visuospatial and memory abilities (mirror neurons), as well as mathematical operations processing^26^. Moreover, as shown by Sowell et al., the parietal lobe presents the most important changes during adolescence, a period of rapid cerebral development^27^.

Structural connectivity was further explored by measuring local graph metrics for each brain region. With a preponderance of the parietal and temporal lobes, significant associations were observed with most of the cortical nodes. In particular, metrics measured in the left precuneus showed a better network integration in relation with FSIQ, mostly driven by the PRI. This result is in agreement with previous studies, highlighting the importance of the precuneus in visuospatial abilities and self-consciousness^28^. Local metrics, measured in the left middle and superior temporal networks, were associated to VCI. These findings are concordant with the report of Crinion et al., showing that speech comprehension employs both temporal lobes, with a left predominance^29^. All these regions have been demonstrated to be related to intelligence as they are included in the P-FIT theory by Jung and Haier^1^. Moreover, in agreement with the report of Basten et al. we observed a relation between PRI and local graph metrics in the subcortical networks^2^. High PRI was associated to high network integration of the right caudate nucleus, region also involved in reasoning^30^.

Our analysis was completed by the characterization of WM fiber-bundles microstructure. Derived from the diffusion tensor, FA values (reflecting WM integrity) and AD (quantifying WM connectivity) could be influenced by several microscopic factors, such as myelination and axonal density or diameter^31,32^. Indeed, positive correlations were observed between both FA and AD values, and IQ subscales in most of the large WM fiber-bundles of both hemispheres. These correlations were mostly located in the commissural and associative fiber-bundles. Particularly, diffusivity metrics were correlated with all intelligence scores (except the PSI) in the uncinate bundle, implied in memory, language, and emotion processing^33^, and the inferior fronto-occipital fascicle, implied in many integrative functions. These findings suggest that high intellectual performances could be related to high axonal myelination level and/or density.

### Methodological limitations

This study presents some limitations. The first one was the low number of participants, related to the difficulty of young children recruitment. Also, the unbalanced number of boys and girls was overcome by including the gender effect in the statistical analysis. This limitation is often encountered in high potential children studies, as girls present the ability of “over-adaptation” which leads to less clinical consultations, thus reducing the number of girls identified as high IQ. While we observed a FSIQ difference between male (128.7 ± 15.2) and females (122.6 ± 21.0), the non-parametric Wilcoxon-test performed for each WISC index between the two gender groups didn’t show any significant difference (e.g. FSIQ: *p* = 0.24). Second, this DTI analysis was performed with 24 directions, that is relatively low compared to current studies. In order to minimize the potential crossing-fibers errors, a spherical deconvolution model was applied instead of classic diffusion tensor model. Finally, because brain WM changes occur continuously during childhood maturation^34^, our results can only be exploited between the ages of eight and twelve years-old. This age range may contribute to the lack of findings in the prefrontal regions. Indeed, the course of brain maturation is known to progress from temporal and occipital towards the parietal and frontal lobes during adolescence, while the prefrontal regions do not mature until the 20 s^35^.

## Conclusion

This study showed significant correlations between several graph metrics based on diffusion measures and psychometric scores. First, the increase of network degree with intelligence scores, confirmed that fiber bundles density of brain networks plays a significant role in children intelligence. Second, considering that modularity represents a measure of segregation level between graph modules, and that transitivity reflects the redundancy in the network connections, the low modularity and low transitivity observed in high IQ children confirmed that homogeneous brain organization correlates with intelligence. These findings show that children’s high IQ may be related to a high fiber density and optimally distributed brain networks, thanks to the global sensitivity of such graph-based methods for the investigation of brain topological organization. Furthermore, diffusion metrics measured in WM fiber-bundles confirmed that children intellectual abilities are correlated with enhanced WM myelination and integrity. In summary, this study demonstrated that intelligence quotient is correlated with the microstructure as well as the topological organization of the WM brain networks.

## Supplementary information

Supplementary Information.

## Data Availability

The datasets generated analyzed during the current study are available from the corresponding author on reasonable request.
