# Supplementary material for: White matter microarchitecture and structural network integrity correlate with children intelligence quotient
Source: Sci Rep. 2020 Nov 26;10:20722. doi: 10.1038/s41598-020-76528-x (PMC7691327; doi:10.1038/s41598-020-76528-x)

**White Matter Microarchitecture and Structural Network Integrity Correlate with Children Intelligence Quotient**

Ilaria Suprano^1#^, Gabriel Kocevar^1#^, Claudio Stamile^1^, Salem Hannoun^2^, Pierre Fourneret^3^, Olivier Revol^3^, Fanny Nusbaum^4^, Dominique Sappey-Marinier^1,5^*

*1. CREATIS, CNRS UMR 5220, INSERM U1206, Université de Lyon, Université Claude Bernard-Lyon 1, INSA-Lyon, France*

*2. Nehme and Therese Tohme Multiple Sclerosis Center, Faculty of Medicine, American University of Beirut, Lebanon*

*3. Service de Psychopathologie du développement de l’Enfant et de l’Adolescent, Hospices Civils de Lyon, France*

*4. Laboratoire Parcours Santé Systémique (EA 4129), Université de Lyon, Université Claude Bernard-Lyon 1 & Centre PSYRENE, France*

*5. CERMEP - Imagerie du Vivant, Université de Lyon, France*

# These authors have contributed equally to this work

**ORCID:** Ilaria Suprano 0000-0002-7940-7406, Gabriel Kocevar 0000-0003-4130-6498, Claudio Stamile 0000-0003-3291-2592, Salem Hannoun 0000-0002-5982-3242, Pierre Fourneret 0000-0002-4606-0362, Olivier Revol 0000-0002-2089-7068, Fanny Nusbaum 0000-0002-0595-3105, Dominique Sappey-Marinier 0000-0003-4104-1514.

**** Corresponding author:* Dominique Sappey-Marinier**

Email: [dominique.sappey-marinier@univ-lyon1.fr](mailto:dominique.sappey-marinier@univ-lyon1.fr)

**Supplementary Materials**

**White matter and gray matter parcellations**

Illinois Institute of Technology (IIT) atlas was used for white matter parcellation, while cortical and subcortical gray matter parcellation was obtained using Desikan atlas. The following Figure S1 shows an illustration of these two atlases. Nifti files are also provided for information.

**Figure S1.** Illinois Institute of Technology (IIT) atlas used for white matter parcellation on the left, and Desikan atlas used for cortical and subcortical gray matter parcellation on the right.


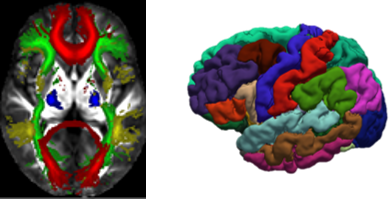

Supplement: Supplementary file 1 — Supplementary Information. [file 41598_2020_76528_MOESM1_ESM.docx]
